# Moral Distress in the Pediatric Intensive Care Unit: An Italian Study

**DOI:** 10.3389/fped.2019.00338

**Published:** 2019-08-13

**Authors:** Patrizio Sannino, Maria Lorella Giannì, Micaela Carini, Mario Madeo, Maura Lusignani, Elena Bezze, Paola Marchisio, Fabio Mosca

**Affiliations:** ^1^Fondazione IRCCS Ca' Granda Ospedale Maggiore Policlinico, Direzione Professioni Sanitarie, Milan, Italy; ^2^NICU, Fondazione IRCCS Ca' Granda Ospedale Maggiore Policlinico, Milan, Italy; ^3^Department of Clinical Sciences and Community Health, University of Milan, Milan, Italy; ^4^ASST Grande Ospedale Metropolitano Niguarda, Bachelor of Nursing, Course Session, Milan, Italy; ^5^Department of Biomedical Sciences for Health, University of Milan, Milan, Italy; ^6^Fondazione IRCCS Ca' Granda Ospedale Maggiore Policlinico, Milan, Italy; ^7^Department of Pathophysiology and Transplantation, University of Milan, Milan, Italy

**Keywords:** moral distress, pediatric intensive care unit, nursing, ethical climate, decision-making, work environment

## Abstract

**Introduction:** There is paucity of data within the Italian context regarding moral distress in intensive pediatric settings. The aim of the present study was to assess the frequency, intensity, and level of moral distress experienced by nurses working in a sample of pediatric intensive care units (PICUs).

**Materials and Methods:** A cross-sectional questionnaire survey was conducted in eight PICUs from five northern Italian regions in a convenience sample of 136 nurses. Moral distress was evaluated using the modified Italian version of the Moral Distress Scale Neonatal–Pediatric Version (MDSNPV). Each item was scored in terms of frequency and intensity on a five-point Likert scale, ranging from 0 to 4. The total frequency and intensity scores for all the 21 clinical items were comprised between 0 and 84. For each item, the level of moral distress was derived by multiplying the frequency score by the intensity score and quantified with a score ranging from 0 to 16. The total score of the moral distress level for the 21 items ranged from 0 to 336.

**Results:** The mean total scores for the frequency, intensity and level of moral distress were 24.1 ± 10.4, 36.2 ± 18.6, and 57.7 ± 37.1, respectively. The clinical situations identified as the major causes of moral distress among nurses in the present study involved end-of-life care and resuscitation. At multivariate logistic regression analysis, number of deaths occurring in PICUs, having children and intention to leave work due to moral distress resulted to be independently associated with a higher total moral distress level.

**Conclusions:** The results of the present study contribute to the understanding of moral distress experience in acute pediatric care settings, including the clinical situations associated with a higher moral distress level, and highlight the importance of sharing thoughts, feelings and information within the multidisciplinary health care professional team for effective shared decision making, particularly in situations involving end-of-life care and resuscitation.

## Introduction

The survival of critically ill children with complex and chronic diseases has significantly improved as a result of advances in pediatric critical care (1). As a consequence, the percentage of survivors with major disabilities has increased (2). Moreover, several other factors may contribute to the complexity of pediatric intensive care units (PICUs) care environments, such as working with an excessive workload and a high patient-operator ratio, the occurrence of poor communication and conflicting opinions within the healthcare team and between the health care team and the family (3–5).

Within this scenario, health care professionals encounter many ethical issues. The concept of moral distress was first defined by Jameton (6) as “a painful psychological imbalance coming from the awareness of the operators about the appropriate choice that cannot be carried out owing to different obstacles impeding the execution.” These obstacles include internal constraints, such as a sense of impotence and a lack of knowledge, as well as external constraints, such as limited communication among team members and between health care professionals and families, a lack of clear definition of roles and responsibilities among the team members, an excessive workload, a lack of agreement among the team members, and scarcity of resources (3–8). Moral distress leads to a variety of problems, including impaired physical and psychological well-being (5, 8, 9). Moreover, job dissatisfaction, burnout and high job turnover have been reported in association with the avoidance of relationships with the patient and/or his/her family (9–13).

Most studies addressing the experience of moral distress among health care professionals have been performed in adult settings (14–16). However, the occurrence of moral distress has also been described in pediatric and neonatal settings (17–21). Within the Italian context, this topic has been addressed in neonatal intensive care units and oncology and hematology wards (17, 18) but it has not been explored in the PICU environment. The study team has previously worked on this subject, reporting low to moderate moral distress among nurses who care for critically ill newborns (17). Lazzarin et al. (18) found moderate to high moral distress among nurses working in pediatric oncology and hematology wards.

The aim of the study was to assess the frequency, intensity and level of moral distress experienced by nurses working in PICUs. Furthermore, we aimed to evaluate the relationship between moral distress and the nurses' demographic characteristics and professional experience.

## Materials and Methods

### Design and Setting

We conducted a cross-sectional questionnaire survey from June to October 2018 at eight PICUs from five northern Italian regions, thus addressing 57% of the 14 PICUs in Northern Italy.

Approval from the institutional review board was obtained. Informed consent was signed by all the study participants.

### Sample

A convenience sample of nurses participated in the survey. The inclusion criteria were nurses working in the PICU. Exclusion criteria were temporary absence from the PICU due to parental leave.

### Data Collection Procedures

Participation in the study was anonymous and voluntary. After contacting the nursing coordinator, the principal researcher organized meetings with the PICU nurses to explain the aim of the study and the correct method for completing the questionnaire. The questionnaire was self-administered and paper -based. Its completion required 10–15 min. The questionnaires were collected 2 weeks after they were distributed.

### Instruments

Moral distress was evaluated using the modified Italian version of the Moral Distress Scale Neonatal–Pediatric Version (MDSNPV), which consists of 21 items that refer to morally distressing situations that nurses experience (13, 17, 22, 23). The Questionnaire items are reported in Table 1.

**Table 1:** Questionnaire items.

**Items**	
1	Provide less than optimal care due to administrative pressures to reduce costs (due to the excessive cost you choose a less expensive alternative to the optimal one)
2	Provide care that gives parents false hopes (e.g., telling of events that occurred in similar situations that could give parents false hopes)
3	Follow the family's wishes to continue life support even though I believe it is not in the best interest of the child
4	Initiate extensive life-saving actions when I think they only prolong dying
5	Follow the family's request not to talk to the dying child about death
6	Carry out the physician's orders to perform tests and treatments that I consider unnecessary
7	Continue to provide care for a hopelessly ill child who is being sustained on a ventilator when no one will make the decision to withdraw support
8	Avoid taking measures when I learn that a fellow nurse or doctor has made a mistake and has not reported it
9	Assist a physician who in my opinion is providing incompetent care
10	Take care of patients for whom I do not feel qualified
11	Assisting students in performing painful procedures on children in order to improve their skills
12	Provide a treatment that does not relieve the child's suffering due to the doctor's fear that a higher dose of drug will cause death
13	Follow the doctor's request not to discuss the child's prognosis with the parents
14	Increase the dose of sedative/opiate in the unconscious child when I think it will accelerate death
15	Do not take action on ethical issues when some staff member or someone in an authority position asks me not to do so
16	Follow the family's desire for child care, but disagree with them, and do so just because of legal concerns
17	Work with nurses or other care providers whom I do not consider competent
18	Witness diminished patient care quality due to poor team communication
19	Ignore situations where parents have not been given adequate information to ensure informed consent
20	Detecting the suffering of the child due to a lack of continuity of care
21	Work with staffing levels of nurses or other care providers that I consider unsafe

Each item was scored in terms of frequency (how often the situation is experienced) and intensity (the degree of the distress caused by the situation) on a five-point Likert scale ranging from 0 (never) to 4 (very frequently) for the frequency scale and from 0 (none) to 4 (great extent) for the intensity scale. The total frequency and intensity scores for the 21 clinical items were comprised between 0 and 84. For each item, the level of moral distress was derived by multiplying the frequency score by the intensity score and quantified with a score ranging from 0 to 16. The total score of the moral distress level for the 21 items ranged from 0 to 336. A mean score for frequency, intensity, and level of moral distress was then calculated by dividing the total scores obtained by 136, that is the number of nurses who completed the questionnaire. As previously reported (16), the frequency and the intensity of moral distress for each item were then categorized on a Likert scale (0–4) as low (0–1.33), moderate (1.34–2.67), and high (2.68–4). The moral distress level was ranked as low (0–1.77), moderate (1.78–7.13), and high (7.14–16).

Data regarding demographic characteristics (age, gender, marital status, and education level) and professional experience (working shift, specialization and professional title, years in the PICU), PICUS characteristics (type of PICU, nurse staffing, nurse to patient ratio, number of beds, number of admissions and deaths during the year preceding the study) were also collected. The participants were also required to indicate whether they intended to leave their current position because of moral distress and/or because of other reasons.

### Analysis

Data are expressed as mean ± standard deviation or number of observations (percentage).

The association between the mean moral distress level scored at each item and the investigated continuous variables (age, length of nursing experience, and length of nursing experience in the PICU) was assessed using the non-parametric bivariate correlation (Spearman test).

The mean moral distress level scored at each item was compared among females vs. males; married vs. single/divorced, having children vs. not having children, working on 24 h shifts vs. other types of shifts, intending to leave the job due to moral distress vs. not intending to leave using the Mann-Whitney U-test.

A multivariate logistic linear regression analysis was then conducted in order to identify which parameters (demographic characteristics, professional experience, intention to leave work due to moral distress, PICUs characteristics in terms of number of admissions, number of deaths, number of beds, and nurse-child ratio) were independently associated with the total score of moral distress level.

Statistical significance was set at the α = 0.05 level. The statistical analyses were performed using SPSS (version 12, SPSS, Inc., Chicago, IL).

## Results

A total of 230 questionnaires were distributed, and 136 (59.1%) were returned. The majority of the participants were female and married, and one out of two had children. The participants' basic characteristics are shown in Table 2. PICUs' characteristics, including the number of respondents in each NICU, are shown in Table 3.

**Table 2:** Basic characteristics of the participants.

**Characteristics**		***N* (%)**
Gender	Female	110 (80.9)
	Male	26 (19.1)
Marital status	Single	38 (28)
	Divorced	15 (11)
	Married	83 (61)
Having children		68 (50)
Working shift	Days (defined as a work schedule during the daytime, either including or not weekends)	20(14.7)
	Rotating	116(85.3)
Specialization (master/master's degree)		23 (17.2)
Registered nurse		95 (69.8)
Pediatric nurse		41 (30.2)
Nurses with the intention to leave work due to moral distress		34 (25%)
Nurses with the intention to leave work due to other reasons		47 (34.6%)
		**Means** ± **SD**
Age (y)		38 ± 8.6
Professional experience (y)		14 ± 9.1
Professional experience in the PICU (y)		10 ± 7.7

**Table 3:** Enrolled PICUs' characteristics.

	**Nurse staffing (*n*)**	**Respondents to survey n (%)**	**PICUs beds (n)**	**Nurse-Child ratio**	**Type of PICU**	**Annual admissions (*n*)**	**Annual deaths (*n*)**
PICU 1	33	17 (51)	2	1:3	Medical and surgical	110	1
PICU 2	14	12 (86)	5	1:2	Medical and surgical	440	3
PICU 3	23	11 (48)	8	1:3	Medical and surgical	480	20
PICU 4	21	20 (95)	6	1:2	Medical and surgical	323	3
PICU 5	55	21 (38)	16	1:2	Medical and surgical including cardiac surgery	1,000	10
PICU 6	25	20 (80)	8	1:2	Cardiac intensive care unit	500	3
PICU 7	42	24 (57)	16	1:2	Medical and surgical including cardiac surgery	600	30
PICU 8	23	11 (48)	6	1:2	Medical and surgical including cardiac surgery	450	25

### Frequency, Intensity, and Level of Moral Distress

The mean total scores for the frequency, intensity and level of moral distress were 24.1 ± 10.4, 36.2 ± 18.6, and 57.7 ± 37.1, respectively. The five clinical situations that scored the top mean values for frequency, intensity and level of moral distress are shown in Table 4.

**Table 4:** Frequency, intensity, and level of moral distress according to each reported clinical situation.

**Rank**	**Item**	**Mean ± SD**
**TOP FIVE ITEMS–FREQUENCY (F) OF MORAL DISTRESS**		
1	Work with staffing levels of nurses or other care providers that I consider unsafe	2.03 ± 1.48
2	Continue to provide care for a hopelessly ill child who is being sustained on a ventilator when no one will make the decision to withdraw support	1.94 ± 1.23
3	Initiate extensive life-saving actions when I think they only prolong dying	1.84 ± 1.12
4	Follow the family's wishes to continue life support, even though it is not in the child's best interest	1.66 ± 0.98
5	Carry out the physician's orders to perform tests and treatments that I consider unnecessary	1.46 ± 1.03
**TOP FIVE ITEMS–INTENSITY (I) OF MORAL DISTRESS**		
1	Continue to provide care for a hopelessly ill child who is being sustained on a ventilator when no one will make the decision to withdraw support	2.43 ± 1.32
2	Initiate extensive life-saving actions when I think they only prolong dying	2.38 ± 1.26
3	Follow the family's wishes to continue life support even though I believe it is not in the best interest of the child	2.18 ± 1.20
4	Assist a physician who in my opinion is providing incompetent care	2.10 ± 1.28
5	Witness diminished patient care quality due to poor team communication	2.0 ± 1.32
**TOP 5 ITEMS–LEVEL OF MORAL DISTRESS**		
1	Continue to provide care for a hopelessly ill child who is being sustained on a ventilator when no one will make the decision to withdraw support	5.84 ± 5.2
2	Initiate extensive life-saving actions when I think they only prolong dying	5.29 ± 4.88
3	Follow the family's wishes to continue life support even though I believe it is not in the best interest of the child	4.26 ± 3.69
4	Witness diminished patient care quality due to poor team communication	3.64 ± 3.89
5	Work with staffing levels of nurses or other care providers that I consider unsafe	3.58 ± 4.08

Among the clinical situations that were indicated as causing a high frequency of moral distress, “Working with nurses or other care providers whom I do not consider competent and who will put the patient's safety at risk” received the highest score (2.03). The clinical situation “Continue to participate in care for a hopelessly ill child who is being sustained on a ventilator when no one will make the decision to withdraw support” received the highest scores for both the intensity (2.43) and level (5.84) of moral distress.

In Table 5 the percentage of subjects who scored ≥3 for moral distress frequency and intensity and ≥8 for moral distress level at each item is reported. A negative weak correlation was found between age, length of nursing experience, length of nursing experience in the PICU and level of moral distress for item 10 “Take care of patients for whom I do not feel qualified” (*r* = −0.25, *p* = 0.002; *r* = −0.25, *p* = 0.003; *r* = −0.30, *p* < 0.0001, respectively) and item 11 “Witness medical students performing painful procedures on patients solely to increase their skill” (*r* = −0.21, *p* = 0.012; *r* = −0.18, *p* = 0.03; *r* = −0.21, *p* = 0.01, respectively). Females reported higher level of moral distress than males for item 8 “Avoid taking measures when I learn that a fellow nurse or doctor has made a mistake and has not reported it” (2.6 ± 3.0 vs. 1.1 ± 1.87, *p* = 0.016), item 10 “Take care of patients for whom I do not feel qualified” (3.5 ± 5.2 vs. 1.6 ± 2.8, *p* = 0.005) and item 11 “Witness medical students performing painful procedures on patients solely to increase their skill” (2.6 ± 2.9 vs. 1.2 ± 1.7, *p* = 0.25). Nurses with children reported higher level of moral distress than nurses without children for item 2 “Provide care that gives parents false hopes” (3.4 ± 3.3 vs. 2.5 ± 3.35, *p* = 0.03). Nurses who stated they intended to leave their work due to moral distress reported higher moral distress level for item 1 “Provide less than optimal care due to administrative pressures to reduce costs” (3.6 ± 4.2 vs. 1.7 ± 2.3, *p* = 0.011) and item 2 “Provide care that gives parents false hopes” (4.2 ± 4.1 vs. 2.5 ± 3.0, *p* = 0.011). No significant association was found with marital status and working shifts.

**Table 5:** Percentage of subjects who scored ≥3 for moral distress frequency and intensity and ≥8 for moral distress level at each questionnaire item.

**Item**	**Frequency**	**Intensity**	**Level**	**Item**	**Frequency**	**Intensity**	**Level**
*N* = 1	12	25	8	*N* = 12	9	36	10
*N* = 2	9	27	0	*N* = 13	4	25	2
*N* = 3	16	39	21	*N* = 14	1	15	1
*N* = 4	25	46	29	*N* = 15	4	24	4
*N* = 5	4	20	3	*N* = 16	3	16	3
*N* = 6	15	26	16	*N* = 17	13	37	15
*N* = 7	31	54	32	*N* = 18	11	37	16
*N* = 8	4	34	7	*N* = 19	4	24	5
*N* = 9	11	42	12	*N* = 20	3	32	8
*N* = 10	7	34	13	*N* = 21	14	41	16
*N* = 11	10	29	10				

At multivariate logistic regression analysis, number of deaths occurring in PICUs, having children and intention to leave work due to moral distress resulted to be independently associated with a higher total moral distress score (Table 6).

**Table 6:** Association among demographic characteristics, professional experience, intention to leave work due to moral distress, PICUs characteristics and total score of moral distress level (multivariate logistic regression analysis).

	**Unstandardized beta**	**CI 95%**	***p***
Hospital admissions (*n*)	0.004	−0.04; 0.05	0.8
Hospital beds (*n*)	−0.16	−0.88; 0.55	0.6
Nurse staffing (*n*)	0.16	−0.61; 0.95	0.6
Nurse-child ratio (1:3 vs. 1:2)	0.24	−19.5; 20.08	0.9
Deaths (*n*)	0.85	0.21; 1.48	0.009
Gender (female vs. male)	6.08	−9.95; 22.11	0.4
Age (years)	−1.42	−3.19; 0.34	0.1
Working shift (day vs. rotating)	5.76	−12.96; 24.50	0.5
Having children (yes vs. no)	15.4	0.09; 30.75	0.049
Marital status (single or divorced vs. married)	12.12	−1.86; 26.11	0.08
Specialization (yes vs. no)	6.62	−10.50, 23.74	0.4
Professional experience (years)	0.35	−1.45; 2.15	0.7
Professional experience in PICU (years)	0.47	−0.76; 1.72	0.4
Intention to leave work due to moral distress (yes vs. no)	19.8	6.03; 33.60	0.005

## Discussion

The present findings indicate that the enrolled nurses working in pediatric intensive care exhibited a low-moderate moral distress assessed through the MDSNPV tool, as indicated by the top five scores reported for the frequency (values ranging from 1.46 to 2.03), intensity (values ranging from 2 to 2.43), and level of moral distress (values ranging from 3.58 to 5.84). It can be hypothesized that, in the investigated PICUs, nurses and physicians might have implemented strategies to alleviate moral distress and promote nurses' work engagement (16, 18, 20), which have led to a good ethical climate. Indeed, Lamian et al. (24) described that high levels of moral distress are associated with poor ethical climate of PICU environments and low levels of collaboration between nurses and physicians.

Trotochaud et al. (25) conducted a survey on moral distress experience in a PICUs, including a sample of 577 nurses. The authors found that the interviewed nurses experienced an average level of moral distress consistent with that obtained in the present study, with values ranging from 4.09 to 5.37. Moral distress experience was investigated by Sannino et al. (17) in 15 level III neonatal intensive care units and a sample of 472 nurses. The top five situations reported by the authors were similar to those found by Trotochaud et al. (25), with moral distress level scores ranging from 4.49 to 5.73.

Consistent with data previously reported in the literature (20, 25–27), the situations identified as the major causes of moral distress among nurses in the present study involved end-of-life care and resuscitation (items 3, 4, 7). Accordingly, Austin et al. (28) reported that moral distress in nurses arises from the conflict between the need to follow physicians' prescriptions aimed at caring for and trying to save the child's life and the struggle to guarantee a dignified death to the patient. Moreover, it has been reported that during end-of-life assistance, nurses seem to perceive themselves as performers and feel less involved in the process of caring for the child and his/her family (10, 20, 29).

In line with previous published data (30, 31), in the present study females experienced a significantly higher level of moral distress than males with regard to items of the questionnaire focusing on team communication (item 8, 11) and nurses' competency in taking care of patients (item 10). This finding can be partially explained by the fact that women tend to experience more emotionally sensitive situations, and, as a result, their level of distress may increase. However, it has to be taken into account that nursing is largely a female profession (32, 33).

In this study, a negative moderate correlation was found between age, length of nursing experience, length of nursing experience in the PICU and level of moral distress for the items “Take care of patients for whom I do not feel qualified to care” and “Witness medical students performing painful procedures on patients solely to increase their skill.” This result suggests that being young and having less nursing experience may be associated with higher moral distress levels, at least in some clinical situations. Accordingly, Abou Hashish et al. (34) reported that younger nurses (<30 years) and those with <5 years of nursing experience were more likely to exhibit turnover intention than older nurses (>30 years) with >5 years of nursing experience. The authors suggested that these results may be partially explained by the association of shift work with a high workload and economic dissatisfaction (34).

In the present study, intention to leave the current job was independently associated with a higher level of moral distress. The experience of a high level of moral distress is widely recognized as a significant contributor to job dissatisfaction, leading to the intention of work leaving (26, 27). Accordingly, Trotochaud et al. (25) reported that 10.3% of the enrolled nurses wanted to leave their job position due to moral distress. Whitehead et al. (27) found that nurses who were considering leaving their job had a higher moral distress score than those who were not considering leaving. Abdomaleki et al. (35), who conducted a survey in emergency departments, reported that, when nurses experience stress, they are more likely to quit their jobs until they are pushed to give up nursing. The number of deaths occurring in PICs was also positively associated with an increased moral distress level. Indeed, the high mortality that characterizes the PICUs clinical setting, especially if associated with an extended illness with multiple hospitalization, may contribute to moral distress occurrence (8). Moreover, it has to be considered that nurses working in PICUs have to face not only the patient's death but also the grief of the family members (4). In the Italian context, Lazzarin et al. (18) reported that 13.7% of the investigated sample left their work position by moving to another ward or hospital because of moral distress, and these nurses experienced a mean total score of moral distress level 60.88 points higher than that of nurses who did not abandoned their position (282.09 vs. 221.21, respectively). Moreover, one out of 4 of the nurses enrolled in the present study stated that they intended to leave their jobs due to moral distress. This result could be partially explained by the specific characteristics of the clinical setting of the study. Nurses working in PICUs care not only for the child's needs but also of those of the child's family, who may have conflicting ideas about the therapeutic path adopted by nurses (7, 8). Accordingly, specifically in regard to the pediatric patient, accepting the family's wishes to provide continuous life support when nurses think doing so is not in the child's best interest has been reported as one of the major causes of moral distress (36). Santos et al. (36) describe the decision-making process in pediatric care as “complex because it involves intermediaries or *proxies*- parents or guardians,” underlining that the convergence of beliefs and values between the team, family and child may not be always obtained.

Team members within the PICU environment need to be encouraged and supported through organizational issues to overcome fears and avoid intemperate personal attitudes when faced with situations that they consider unethical and contrary to their perception of what is right or wrong (36). Davidson et al. (37) reported that meetings between the team and the family improve communication and parent satisfaction and reduce the conflicts that may arise from the different perspectives regarding to the therapeutic path to be taken. Good communication among the family and the health care team improves reflection and information sharing to promote effective and shared decisions (37, 38).

The study presents some limitations. First, the data collected relate only to 59.1% of the nurses who were invited to complete the survey, and consequently, these results are not applicable to the entire population. Second, several PICU' characteristics, such as the percentage of withdrawal of therapies and the number of conflicts experienced in the year preceding the study have not been collected. Moreover, the administration to the enrolled nurses of the question regarding the intention to leave their current position due to moral distress may represent an additional potential bias whereas, on the other hand, no investigation about other potential causes of moral distress in nurses' professional and personal lives has been performed. Lastly, the potential bias caused to the enrolled nurses by the principal researcher during initial explanation of the aim of the study and the potential for contamination if nurses conferred with others during the 2 weeks of finishing up the survey have not been taken into account in the data analysis.

The results of the present study contribute to the understanding of moral distress experience in acute pediatric care settings, including the clinical situations associated with a higher moral distress level, and highlight the importance of sharing thoughts, feelings and information within the multidisciplinary health care professional team for effective shared decision making, particularly in situations involving end-of-life care and resuscitation. Further larger additional studies are needed in order to implement tailored strategies aimed at reducing the burden for reducing the burden of moral distress in acute pediatric care settings.

## Data Availability

The datasets generated for this study are available on request to the corresponding author.

## Author Contributions

PS and MG conceived and designed the study, and wrote the article. MC, MM, and EB collected the data and were responsible for database management. ML, PM, and FM contributed to the discussion of the results and provided suggestions concerning the content and concept of the article.

### Conflict of Interest Statement

The authors declare that the research was conducted in the absence of any commercial or financial relationships that could be construed as a potential conflict of interest.
